# The Social Media Industry as a Commercial Determinant of Health

**DOI:** 10.34172/ijhpm.2022.6840

**Published:** 2022-04-27

**Authors:** Marco Zenone, Nora Kenworthy, Nason Maani

**Affiliations:** ^1^Faculty of Public Health & Policy, London School of Hygiene and Tropical Medicine, London, UK.; ^2^School of Nursing and Health Studies, University of Washington Bothell, Bothell, WA, USA.

## Introduction

 Social media has formed a key area of concern for public health. In recent years, elections across the world, the Black Lives Matter protests, and the coronavirus disease 2019 (COVID-19) pandemic have highlighted major social media platforms’ roles in amplifying and inadequately moderating mis/disinformation, racism, sexism, and xenophobia. There is growing attention to vested interests, such as health-harming industries, utilizing social media targeted marketing opportunities to promote their products and shape public and political discourse.^1^ Mental health concerns associated with social media use, such as body image issues,^2^ are also increasingly reported. Taken together, social media can have direct impacts on users and indirect impacts to societies by undermining key determinants of health.

 Unfortunately, social media-related public health concerns are often attributed to the decisions or actions of users or considered by-products of platform usage. The role of social media platforms themselves, and the companies that design them, is rarely considered. In many cases health research treats platforms as a tool for gathering data to investigate online trends, or as a partner for carrying out research or interventions. These approaches do not allow us to understand the design and purpose of such platforms themselves as drivers of health outcomes, or the potential conflicts of interest between public health and social media companies.

 Social media companies are reliant upon advertising for revenue, thus, they prioritize strategies that retain user attention to increase advertising opportunities, often deploying algorithms to suggest content specific to users’ interests. However, critics argue that the algorithms underpinning content promotion may contain or lead to harms, and that social media companies may be aware of the harms, but do not act on them because they are profitable. Summarized by Meta (then Facebook) whistleblower Frances Haugen: “Facebook has realized that if they change the algorithm to be safer, people will spend less time on the site, they’ll click on less ads, [and] they’ll make less money.”^3^

 We argue in this viewpoint that social media platforms’ business and political practices are consistent with the characterizing features of the commercial determinants of health (CDOH). Kickbusch and colleagues define the CDOH as “the strategies and approaches used by the private sector to promote products and choices that are detrimental to health.”^4^ Here, we outline the predominant reasons to recognize social media producers as a CDOH. In making this case we draw on Meta as our primary example, given the widespread use of its platforms (Facebook, Instagram, and WhatsApp) while also recognising the diversity of companies, actors and platforms that comprise current social media landscapes.

## Reasons to Recognize the Social Media Industry as a Commercial Determinant of Health

###  Addictive Platform Design Features Are Associated With Mental Health Consequences

 According to former social media developers and social scientists, platforms owned by Meta such as Facebook developed their interfaces, algorithms, and features to elicit addictive behaviors and retain user attention for advertisement views.^5^ The addictive features of the platform, such as “endless scrolling,”^6^ and subsequent high user engagement, have led to associations of social media use with mental health symptoms or distress, particularly among young people.^7^ Social media platforms refute linkages between their business practices and public health concerns, while seeking to hide evidence about the potential harms of their platform. For example, in September 2021, an investigative report by the Wall Street Journal found that Meta (then Facebook), which owns Instagram, conducted research showing that Instagram usage is associated with negative body image, particularly among girls and young women, but kept these findings hidden and downplayed the risk to the public.^8^

###  Amplification and Proliferation of Dis/Misinformation and Forms of Hate Speech

 Social media platforms enable the spread of misinformation and forms of hateful speech, rhetoric, or polarizing content through limited content moderation^9^ and algorithms that match users to content, groups, or pages based on their interests or previous media viewed, to maximize engagement and advertising revenue.^10^ This creates “echo chambers” exposing users to content that reinforces their perspectives or confirmation biases.^11^ Advocates have drawn attention to the limited efforts made to prevent the spread of content with misinformation or hateful sentiment in algorithm-promoted or recommended content^3^ – thus, if a user is viewing misinformed or hateful content, platforms such as Facebook may suggest additional, related content. Misinformation and its associated difficulties for public health officials have been demonstrated during the COVID-19 pandemic, which the World Health Organization (WHO) labelled an “infodemic.”^12^ Avaaz – a United States non-profit activism group – released a report that found that misinformation related to vaccines, masking, and social distancing was viewed approximately three billion times on Facebook by July 2020.^13^ It has been claimed that spread of disinformation has also incited or excused violence. The military organizers of the Rohingya genocide in Myanmar allegedly used Facebook as “a tool for ethnic cleansing” through anti-Rohingya disinformation campaigns.^14^

###  Research Control and Funding

 Many social media platforms implement strict controls on their data for research purposes. To access data, researchers must often apply for access, stating their personal information for consideration, whereupon platforms can choose whether to grant access. For example, Meta has offered research tools such as CrowdTangle. After access is granted, Meta limits information collection and restricts access to key metrics such as reach or impressions. This creates significant, arguably intentional, difficulties in monitoring social media trends and activity. The New York Times reported that a former CrowdTangle overseer left Facebook because the “company does not want to invest in understanding the impact of its core products” and “doesn’t want to make the data available for others to do the hard work and hold them accountable.”^15^ Publicly available tools, such as the Meta Ad Library, offer only basic information. Meta does not allow scraping on their platform using tools outside their public tools and can withhold or remove access to research tools which likely has a dampening effect on what researchers can study and publish. In July 2021, Facebook removed the accounts of misinformation researchers who were scraping political advertising content, which is unauthorized by the platform.^16^ Social media platforms also fund research opportunities related to public health concerns exacerbated by their platforms, such as misinformation.^17^ The large and growing body of evidence of other health-harming industries such as the sugar lobby concealing and funding research advancing their interests, or ceasing research that might be harmful to revenue,^18^ should be an important caution and potential conflict of interest in such training or funding programs.

###  Targeted Marketing Opportunities and Surveillance Data

 Social media platforms provide powerful targeting tools and data for businesses, special interest groups, and politicians to target defined demographics with business or political ads. Meta allows targeting of specific cities, communities, ages, genders, education levels, job titles, interests, and consumer behaviors (previous purchases made). Many health-harming industry actors, such as alcohol companies, use social media platforms to promote their products to defined groups. There are voluntary or legal commitments across such industries and by most social media platforms to not target specific groups (such as children) and prohibit the marketing of certain products (tobacco).^19^ However, these restrictions are demonstrably circumvented^20,21^ and promotion still occurs through direct-to-consumer marketing, peer-to-peer marketing, or influencer marketing. Industries may also utilize social media for political and social purposes related to the regulation of their products, such as preventing taxes on certain products including sugar-sweetened drinks, where content in advertisements may be misleading due to lack of Facebook oversight on truthfulness in political advertising.^1^ Finally, social media platforms may sell data, which is then used by health-harming industries for advertising purposes.

###  Coalition Building With Health Organizations

 Social media platforms invest in developing relationships with health-related organizations across the world. Notably, during the COVID-19 pandemic, the WHO announced they were partnering with Meta platforms and Viber to deter misinformation and deliver evidence-based information to the global public.^22^ Meta (then Facebook) provided the WHO $120 million in advertising credit to correct misinformation. The relationship with the WHO and the specific focus on addressing misinformation spread is paradoxical. Meta’s platforms, business decisions, and algorithms are in part responsible for the spread of misinformation about COVID-19 – including its failure to ban anti-vaccination advertisements until approximately seven months after the pandemic declaration.^23^ The promised actions taken by Meta – including removing specific kinds of content and promoting science-informed information – do not change the root causes of misinformation spread. Indeed, addressing the root causes of these issues, such as algorithms and lack of enforced content moderation, would likely negatively impact Meta’s business model. Thus, partnering with the WHO enables Meta to receive positive publicity without risk to their revenue streams or threat of external regulation.

###  Corporate Social Responsibility Initiatives

 Similar to health-harming commodities industries, social media platforms utilize corporate social responsibility (CSR) initiatives to advance their interests. A notable CSR initiative undertaken by Meta is ‘Internet.org,’ which was later rebranded into the ‘Free Basics’ program, which brings basic-level internet connectivity to countries or areas with low internet access, arguing it is necessary for economic development.^24^ However, the program primarily lets users access the internet through Facebook and Meta-related products, thus introducing controversy over potential gatekeeping of the internet and further exposure to misinformation. Additionally, despite being represented as a philanthropic endeavour, Meta stands to profit from the recruitment of billions of persons not yet accessing the internet. The Free Basics program is heavily criticized – described as “digital colonialism”^25^ and accused of “us[ing] disadvantaged populations and unregulated territories for digital experiments and data extraction.”^24^ As of July 2019, there are 28 African countries currently accessing the Free Basics Program.

###  Promotion of Self-regulation Discourse

 In response to the calls to regulate social media platforms due to the rapid spread of misinformation during the COVID-19 pandemic and recent elections, platforms such as Facebook announced voluntary actions to appease critics and limit regulatory interventions. Initiatives of note include investing in third-party fact-checking services and displaying warnings or removing content proven to be misleading or false. In 2020, Meta (then Facebook) also launched the ‘Oversight Board’ – which it described as an independent panel to determine whether content removal decisions are justified, though still under their funding control. Commenting on the future of Section 230 in the United States – the policy that shields social media platforms from legal liability for content posted by their users – Zuckerberg argued that “[i]nstead of being granted immunity, platforms should be required to demonstrate that they have systems in place for identifying unlawful content and removing it. Platforms should not be held liable if a particular piece of content evades its detection.”^26^ Facebook has most of the systems mentioned already implemented, yet as many point out, these systems have failed to adequately address the social harms of its platforms.^9^

## Conclusion and Future Research

 This article argues to recognize the social media industry a CDOH due to the direct and indirect consequences of their products and actions. Platforms directly impact users’ health through their products, which are associated with mental health concerns and contain addictive features. The products offered by social media platforms, such as targeted advertising, are more powerful than traditional mechanisms for which regulations were developed. Health harming industries can reach targeted audiences with specific messaging to sell products or protect their interests. The structure of social media, including user data collection, and subsequent ad targeting, compromises individual agency.^27^ Indirect health consequences such as the spread of dis/misinformation, erosion of democratic values and processes, and third-party data access and surveillance, negatively impact broader determinants of health. This context suggests a conflict of interest between social media platforms’ profits and public health, demonstrating the need for social media industry regulation. However, limited transparency from social media platforms, regarding issues like the data they collect and how it is used, makes regulation difficult.^28^ This is exacerbated by the deployment of strategies common to other industries, such as controlling data availability, building coalitions, using CSR, and promoting self-regulation. The similarities between the social media industry and other health harming industry strategies to protect profits underscore the need to develop a cohesive systems approach across industries^29^ and adopt integrated, rather than siloed, regulation strategies.^30^ The lack of regulation of social media enables other industries to abuse such platform tools, amplifying the public health concerns.

 Moving forward, we encourage public health researchers, and particularly those using social media in their research, to carefully assess the complex health impacts of social media technologies and the nature of the business structures underpinning them. The reasons we outline to consider social media a CDOH are introductory and further exploration is warranted. Future research is needed to analyze social media corporate political activity, specifically lobbying activities, research funding and influence, data surveillance practices, coalition building with trade groups or health-related organizations, and social media expansion activities in low- and middle-income countries in order to identify effective governance strategies. In summary, we urge recognition of, and further research on, social media companies as a key CDOH of health in the 21st century.

## Acknowledgements

 The authors kindly thank Prof. Mark Petticrew, Dr. Benjamin Hawkins, and Dr. May van Schalkwyk for their feedback, guidance, and suggestions. The authors also thank the manuscript reviewers for their insightful and helpful comments.

## Ethical issues

 Not applicable.

## Competing interests

 Authors declare that they have no competing interests.

## Authors’ contributions

 All authors contributed to manuscript conceptual design. MZ wrote the manuscript. All authors edited and approved the manuscript.
